# Widespread State-Dependent Shifts in Cerebellar Activity in Locomoting Mice

**DOI:** 10.1371/journal.pone.0042650

**Published:** 2012-08-03

**Authors:** Ilker Ozden, Daniel A. Dombeck, Tycho M. Hoogland, David W. Tank, Samuel S.-H. Wang

**Affiliations:** 1 Department of Molecular Biology, Princeton University, Princeton, New Jersey, United States of America; 2 Neuroscience Institute, Princeton University, Princeton, New Jersey, United States of America; 3 Lewis-Sigler Center for Integrative Genomics, Princeton University, Princeton, New Jersey, United States of America; Tokyo Medical and Dental University, Japan

## Abstract

Excitatory drive enters the cerebellum via mossy fibers, which activate granule cells, and climbing fibers, which activate Purkinje cell dendrites. Until now, the coordinated regulation of these pathways has gone unmonitored in spatially resolved neuronal ensembles, especially in awake animals. We imaged cerebellar activity using functional two-photon microscopy and extracellular recording in awake mice locomoting on an air-cushioned spherical treadmill. We recorded from putative granule cells, molecular layer interneurons, and Purkinje cell dendrites in zone A of lobule IV/V, representing sensation and movement from trunk and limbs. Locomotion was associated with widespread increased activity in granule cells and interneurons, consistent with an increase in mossy fiber drive. At the same time, dendrites of different Purkinje cells showed increased co-activation, reflecting increased synchrony of climbing fiber activity. In resting animals, aversive stimuli triggered increased activity in granule cells and interneurons, as well as increased Purkinje cell co-activation that was strongest for neighboring dendrites and decreased smoothly as a function of mediolateral distance. In contrast with anesthetized recordings, no 1–10 Hz oscillations in climbing fiber activity were evident. Once locomotion began, responses to external stimuli in all three cell types were strongly suppressed. Thus climbing and mossy fiber representations can shift together within a fraction of a second, reflecting in turn either movement-associated activity or external stimuli.

## Introduction

Most information processing in the cerebellum takes place through the interplay of its two major excitatory inputs, the mossy fiber (MF) and climbing fiber (CF) pathways. The manner in which these pathways converge has led to the suggestion that the cerebellum guides action as a prediction or correction circuit [1]. In this framework, CF-encoded error signals act to modify the effects of MFs, the principal source of ongoing drive to Purkinje cells (PCs) and to the deep nuclei, the cerebellum's ultimate output. CFs fire when events deviate from current expectations, thus shaping MF-driven PC output immediately and, on longer time scales, acting as a teaching signal to instruct plasticity (see [2] for review).

Major aspects of cerebellar circuit dynamics during behavior are unknown. Activity from individual granule cells (GCs), which are excited by MFs, has not been reported from awake, behaving mammals, nor activity from molecular layer interneurons, which are excited by GCs. Under anesthesia, GCs can be near-silent and respond to sensory activation with a brief burst of spikes [3], [4], [5]. MFs can also fire in complex ongoing patterns to represent joint movement [6] and vestibular activity [7]. In awake animals, multiunit GC recordings [8], [9], [10] show elevated activity compared with anesthesia, potentially reflecting drive from the forebrain [11]. Underlying this multiunit activity is a wide variety of possible firing patterns in individual GCs.

A major indication that cerebellar responsiveness is regulated by behavioral state comes from complex spike recording from PCs (reviewed in [12]). Complex spikes are driven by CF firing, which occurs at ∼1 Hz, and are identifiable by a characteristic electrical waveform and a pan-dendritic calcium transient that can be imaged in vivo [13], [14], [15]. Compared with the resting state, PCs respond to specific limb touch with lower probability during walking, a phenomenon termed sensory gating [16]. Modulations are fast, occurring within a fraction of a second [17].

However, until now it has not been known how behavior-dependent response shifts might be reflected either in MFs, or for that matter, in ensembles of CFs. Ensembles of CFs are of interest because they can fire together with millisecond-level synchrony [18], [19], [20], driven by incoming excitatory drive to gap junction-coupled olivary neurons [21], [22], [23]. In PCs less than 100 microns apart, this synchrony can be very high [15], [24], [25], [26] because neighboring CFs emanate from neighboring olivary neurons. Synchrony largely does not arise from common innervation, since CF branch terminations are sparse in the cerebellar cortex [27] (see also [15]). Olivary synchrony may convey specific signals to the cerebellar nuclei via olivonuclear excitation and disynaptic PC inhibition converging on deep nuclear targets [28], potentially driving MF-deep nuclear synaptic plasticity [29]. Regulation of CF responses takes place at a location upstream of the inferior olive [30], raising the possibility that coordinated changes in MF activity and CF synchrony may have both immediate and long-lasting downstream effects in the deep nuclei.

Here we report GC, PC and molecular layer interneuron activity using two-photon calcium imaging and extracellular recording from head-fixed mice locomoting on a spherical treadmill. Our report is the first to describe ensemble imaging from GCs and interneurons in awake mice. We investigated patterns of GC and interneuron activity and PC co-activation during locomotion, resting, and in response to external stimuli. Using these approaches we were able to identify rapid, coordinated shifts in MF and CF representations of external stimuli and behavioral state.

## Materials and Methods

### Spherical treadmill and two-photon microscope

Imaging was performed with a custom-built two-photon microscope similar in design to the movable objective microscope (MOM) (Sutter Instruments) integrated with a spherical treadmill as described in detail previously [31], [32], [33]. Briefly, the treadmill, an 8-inch diameter foam ball (Floracraft), was levitated under the microscope objective using a thin cushion of air under the ball emanating from a custom-made casting containing air jets. The air cushion was produced by 8 symmetrically-arranged 0.25-inch diameter holes through the casting, pressurized with air at ∼25 psi. Treadmill rotation was sensed by two optical computer mice (MX-1000, Logitech).

Two-photon microscopy was done using a Ti:sapphire laser (Mira 900 or Chameleon Ultra II, Coherent, 100-fs pulses at 80 MHz) tuned to an excitation wavelength of 880 nm. The microscope had two imaging channels, red and green, with a primary short-pass filter 680 sp (Semrock) and 535/50 and 610/75 emission filters (Chroma) for red and green fluorescence detection, respectively. ScanImage 2.0 [34] was used for microscope control and image acquisition. Images were acquired at 1.1 ms/line through a 40×, 0.8 NA objective (Olympus). Calcium activity was monitored by collecting 1000–2000 images per movie.

### Animal preparation

Experimental procedures were approved by the Princeton University Institutional Animal Care and Use Committee and performed in accordance with the animal welfare guidelines of the National Institutes of Health. The animal was prepared as described previously [31], [32] with the exception of the location of the craniotomy. Mice (male B6CBAF1 or C57/BL6 5–7 weeks old) were anesthetized with isoflurane (0.5–2.0%) and a half-disc craniotomy of 3 mm (full disc) diameter was made over lobule IV/V of vermis. The recording location was confirmed by analyzing the arrangement of cerebellar folia in the fixed brain of the same mouse strain. A two-piece, 1-gram titanium assembly was permanently affixed to the skull over paramedian lobules IV/V of the cerebellum. This assembly held in place a coverslip with a pre-molded Kwik-Sil (World Precision Instruments) plug that exerted slight pressure on the surface of the dura to minimize brain motion during imaging. Imaging was performed 0.25–1.0 mm from the midline.

### Monitoring and characterizing animal movement

During experiments, movements were recorded with a commercial digital camcorder (DCR-SR200, Sony) monitoring the animal from its right side. A set of infrared light emitting diodes was used as the illumination source. Their wavelength (945 nm) did not interfere with two-photon imaging. The animal movies were stored as MPEG movie files (30 ms frame time) and analyzed off-line. To synchronize the animal movies with the two-photon movies, the slow scanning mirror galvanometer signal from the two-photon microscope was fed into the audio channel of the camcorder.

Behavioral states were identified by inspecting the animal movies frame by frame without reference to fluorescence data. Resting was defined as the animal sitting or standing on a motionless treadmill. Locomotion was defined as a coordinated rhythmic movement of all four limbs when all of the paws of the mouse were on or close to the surface of the treadmill. This movement of the mouse caused the treadmill to rotate. Locomotion was often forward motion, but also included sideways and backwards motion. In the analysis of stimulus-triggered responses during locomotion or locomotion triggered responses we used locomotion episodes at least 3 s long and 2 s long, respectively. Time periods in which the animals were resting but the treadmill continued to move after a recent locomotion episode were excluded from data analysis. Movement analysis was restricted to two categories, resting or locomotion. Results obtained using animals familiarized with the treadmill for 2 days were indistinguishable from data from unfamiliarized animals, so data from these groups were pooled.

### Aversive stimuli

Stimuli were applied as airpuffs to the trunk or as clap sounds. Airpuffs were 100 ms long air pulses at 20 psi (Pressure System IIe, Toohey Co.) through a 1 mm diameter tube. Claps were generated using a custom-built wooden clapping tool. A Digidata acquisition system (1322A, Molecular Devices, Sunnyvale, CA) and pClamp software (Clampex 8.2, Molecular Devices) was used to simultaneously record the movements of the treadmill, the timing of the stimuli, and the command voltage for the slow imaging galvanometer in order to align the animal's movements with the two-photon fluorescence data.

### Loading and expression of calcium indicators

For imaging Purkinje cell dendrites and molecular layer interneurons, tissue was bulk-loaded with the calcium indicator Oregon Green BAPTA-1/AM (OGB-1; Invitrogen Corporation) as previously described [13], [31]. Putative granule cells were imaged either with OGB-1 or by inducing the expression of G-CaMP3 [35]. For G-CaMP3 expression, 30–50 nL of a solution containing AAV2/1-synapsin-1-GCaMP3 [33] was injected separately at three depths (250, 275 and 300 µm) with a beveled glass micropipette (∼1–2 MOhm). Imaging experiments were performed when GCaMP3 expression was sufficiently high, after at least two weeks. Compared with the distribution of OGB-1, G-CaMP3 expression showed greater contrast for localizing putative individual GCs.

### Analysis of imaging data

Analysis of imaging data comprised three steps: (1) correction for brain motion [36], (2) identification of units (GCs, PC dendrites or molecular layer interneurons), and (3) extraction of ΔF/F traces for each identified unit and identification of complex spike-associated signals in the case of PC dendrites [14], [26].

### Correction of brain motion artifacts

As previously described [31], two-photon movies recorded from head-fixed awake mobile mice are deformed by brain motion-related artifacts. Previous measurements using fluorescent dye and particles in neocortex demonstrated an absence of significant brain motion above 30 Hz in an awake, treadmill-running mouse [33], [36]. This finding suggested that a correlation-based procedure on video-rate data to calculate the frame-by-frame amount of shift might be able to remove motion-related artifacts in cerebellar fluorescence signals. Residual movement artifacts, which would have appeared as distortions within a single frame, were not seen (**Movie S1**). Thus whole-frame-shift correction is a sufficient approach to motion correction. The same procedure was used for subsequent analysis of interneuron and granule layer cell data.

Our imaging rates (35 ms/frame) and pixel resolution (∼2 µm/pixel) allowed artifacts due to brain motion to be corrected by a 2D cross-correlation-based algorithm as explained in detail previously [33], [37]. This algorithm requires observation of structures with high contrast within the field of view. In some experiments, especially where PC dendrites were imaged, it was necessary to generate fluorescence contrast in the field of view without interfering with functional imaging. For this purpose, Alexa 594 dextran (Invitrogen) was electroporated through a beveled glass pipette (∼4 MΩ when filled with physiological-strength salt solution) at a depth ∼110 µm from the cerebellar surface. Electroporation was accomplished by applying 700–1000 pulses of negative current (4–5 nA, 0.2 ms pulses) through the pipette every 500 ms. Electroporation with Alexa 594 dextran resulted in sparse labeling of a only a few Purkinje cells. In some imaging experiments, these labeled cells were positioned at the edge of the field of view to facilitate correction of brain motion-related artifacts in the image stacks. The Alexa 594 fluorescence spectrum is in the red wavelength region and well separated from the OGB-1 fluorescence spectrum. Therefore, the Alexa 594 dextran fluorescence could be collected through a separate imaging channel (red channel) without affecting the calcium imaging channel (green channel). In experiments with interneurons and granule cells, green channel fluorescence from cell bodies was sufficient for motion correction.

### Identification of neurons

**Figure 1:**
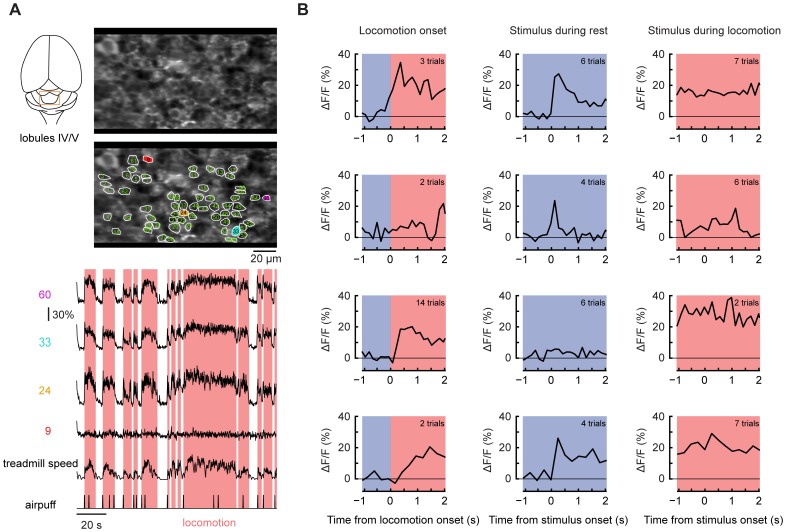
Locomotion- and stimulus-associated signals in granule cell layer neurons recorded using the calcium indicator protein G-CaMP3. (**A**) A field of view showing 62 putative granule cells (GCs) with four example fluorescence traces. The labels at left of traces indicate the neurons monitored in the image. (**B**) Example traces from putative GCs representative of a few selected categories (see **Table 1**) of responsiveness during locomotion and in response to stimuli presented during rest or locomotion: *Row 1*, locomotion-responsive cell responding to stimuli during rest but not running; *Row 2*, locomotion-unresponsive cell responding to stimuli only during rest; *Row 3*, locomotion-responsive cell unresponsive to stimuli at all times; *Row 4*, locomotion responsive cell responding to stimuli at all times. For each row: *left column*, locomotion onset-triggered averages; *center*, stimulus-triggered averages during rest; *right*, stimulus-triggered averages during locomotion. The pink-shaded regions indicate epochs of locomotion identified from video recordings.

Dendrites of PCs were identified by using spatial independent component analysis (sICA) as implemented with the FastICA algorithm [38] applied to brain motion-corrected movies. Interneuron masks were selected manually from motion-corrected movies and were identified by their bright, clearly delineated cell bodies and location above the PC layer. Interneurons that were less brightly labeled or fired at lower rates might not be detected as a result of this procedure. OGB-1-loaded GCs were additionally identified by the occurrence of at least one calcium transient during recording. GCaMP3-labeled GC masks were selected based on morphology: the GCs were identified as ring-like bright structures 4–8 µm wide with dark cores, indicative of excluded nuclear volume (**Figure 1**). Ambiguous structures lacking these features were not analyzed.

### Fluorescence signal analysis

The fluorescence trace of a PC dendrite, molecular layer interneuron or GC was calculated by calculating the mean fluorescence intensity of the pixels contributing to the identified structure, for each frame. The mean intensity values in sequential frames constituted sequential time points in the fluorescence trace. Traces were filtered by the following procedure: At each time point in the fluorescence trace, the fluorescence intensity was divided by a baseline defined, point by point, as the lowest 8% percentile value of the fluorescence values within a time window surrounding that point [32]. The time window was ±0.5 s for PCs and ±12.5 s for GCs and molecular layer interneurons. For many of the neuron fluorescence traces, this method reliably subtracted slow changes without significantly filtering longer events that had distinct onsets and offsets; however, in some of the GC traces in which much longer duration transients were present, this method was found to alter the longer events and hence was not used. Instead, the baseline for these neurons was defined by hand and then subtracted from the raw traces. This procedure yielded a smooth baseline trace that was not influenced by slow background fluctuations or by changes in the rate of spontaneous firing events, allowing the fluorescence value at each point to be normalized to local baseline intensity, F. The net effect was that of a high-pass filter. The quantity ΔF was calculated by subtracting the rolling baseline value and then ΔF/F was calculated.

For triggered averages, a putative GC was classified as responding if its fluorescence intensity in a 0.28 s interval after the stimulus or the locomotion onset increased more than 2 SD above the pre-event mean.

### Detection of complex spike-associated fluorescence signals

ΔF/F traces were used to identify the complex spike-associated calcium transients. Detection of complex spike-induced calcium transients in PCs was developed by validation against data sets containing simultaneous imaging and electrophysiological recordings from the same PC dendrite where each complex spike triggered a calcium transient with a rise time ≤10 ms and decay time of t_1/2_ = 0.17±0.05 s [14]. These calcium events were defined using a metric P, the product of a local 2-point slope and a 3-point integral starting from the second of the 2 points used to calculate the slope. In paired imaging and electrical recording experiments this metric has improved detection over a previous template fitting-based method [14], especially in resolving spike bursts. The event times were defined as points (a) that were greater than noise fluctuations (p<0.05, one-tailed test) and (b) for which P exceeded a threshold that maximized the true event detection rate in experiments with simultaneous imaging and electrophysiology data. Under our conditions, P was determined empirically to be 0.001. This algorithm slightly outperforms template matching (our past approach) for resolving complex spikes separated by intervals of ∼0.1 s. Although this procedure might classify some successive complex spikes as single events, single-unit extracellular recordings from PCs in awake mice did not show significant high-frequency (>10 Hz) complex spike bursting.

### Purkinje cell dendritic co-activation

All correlation values were Pearson correlation coefficients of spike trains. Irrespective of steady-state rate, Pearson correlation coefficients are zero for independent firing sequences. The physical distance between two dendrites was defined as the mediolateral component of the distance between the centers of mass of the two regions of interest. The co-activation traces were calculated by adding all the spike trains within a field of view and dividing it by the number of dendrites. This defined the co-activation value as a fraction, ranging from 0 to 1, of simultaneously active dendrites within an image frame.

The co-activation index, CI, was defined as the fraction of PC dendrites active within a single movie frame. “Co-activation events” were defined occurring in frames where CI >0.35. This value of CI was found to maximize the ratio of co-activation events during locomotion divided by resting.

To identify a break point in the plot of response probability relative to locomotion onset, adjusted CI values were calculated by subtracting CI values averaged from times when no stimuli were given. The deviance on each side of a candidate break point was calculated as the sum-of-squares deviation from the mean adjusted CI [39]. The break point was defined as the time for which the sum of left-side and right-side deviance was minimized. A confidence interval on the location of the break point was determined using half-maximal points surrounding the point of minimal total deviance.

### Electrophysiological recording

Recordings from all cell types were performed with 5–7 MΩ glass pipettes filled with ACSF containing in mM: 150 NaCl, 2.5 KCl, 10 HEPES (pH = 7.4). The recordings were made with an Axoclamp 2B (Molecular Devices), then further amplified and filtered to pass 0.3–10 kHz (Model 440 amplifier, Brownlee). The signals were sampled at 20 kHz (Digidata 1322A) and for further analysis offline. PCs were identified by the occurrence of complex spikes. Recorded units were characterized as interneurons if they were in the molecular layer (as assessed by imaging the location of the recording pipette tip in many experiments) and did not fire complex spikes.

For envelope calculation in GC layer recordings, the raw electrophysiological recording was narrow-band filtered to pass 1400–4200 Hz [9]. The positive frequency components were removed from the Fourier transform of this signal. The signal envelope is the absolute value of the inverse Fourier spectrum of this reduced signal, multiplied by 2 to compensate for the losses associated by the removal of the half of the spectrum. Finally, the low-frequency components of the envelope were calculated by low-pass filtering the signal envelope at 20 Hz.

### Data analysis

All analysis was performed using custom scripts written in MATLAB (Mathworks, Natick, MA). Unless specified otherwise, all values are given as mean ± SD in the text and mean ± SEM in the figures. Statistical tests were one-tailed unless otherwise specified.

## Results

### Imaging calcium dynamics from ensembles of cerebellar neurons in awake mice

We used Oregon Green BAPTA-1/AM (OGB-1) and the genetically encoded indicator G-CaMP3 to monitor calcium changes in the cerebellar cortex using two-photon microscopy in head-fixed mice locomoting on a spherical treadmill [31] (see **Materials and Methods** and **Movies S2** and **S3**). We recorded fluorescence movies from zone A of vermis lobule IV/V (**Figure 1A**), a region that represents forelimb and hindlimb sensory/motor information [40], [41]. We recorded activity in the molecular layer from PC dendrites and interneuron somata, and at greater depth from cells in the GC layer (GCL). The mouse's movements were recorded using an IR-sensitive camcorder and additionally by monitoring treadmill rotation using optical mice placed in close proximity to the ball. Locomotion was defined as repetitive patterns of limb movement that after a delay of less than 0.3 s began to drive treadmill rotation. In this way it was possible to monitor signaling in CF and MF pathways before, during, and after episodes of locomotion and in response to aversive stimuli. Stimuli were either airpuffs delivered to the lateral flank or loud clapping sounds, both intense enough to sometimes evoke a startle response or locomotion. The imaging data in this paper represent a total of 658 granule layer cells (total 7 mice: 285 cells, 2 mice, G-CaMP3 and 373 cells, 5 mice, OGB-1), 75 molecular layer interneurons (3 mice, OGB-1), and 937 PCs (5 mice, OGB-1).

### Individual GC responses during locomotion and in response to aversive stimuli

**Figure 2:**
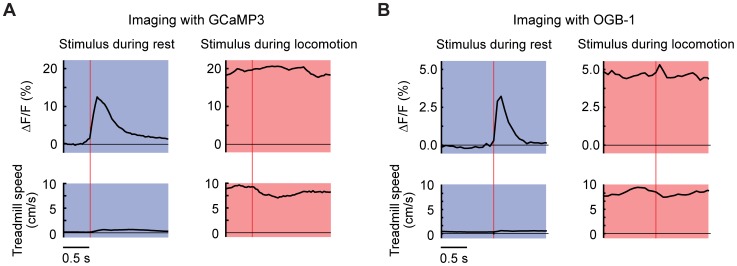
Locomotion-dependence of responses in imaged putative cerebellar granule cells. (**A**) Averaged across all cells imaged using GCaMP3, stimulus-triggered fluorescence average in resting mice (top left) and during locomotion (top right). Bottom, treadmill speed averages. Vertical lines indicate stimulus time. (**B**) The same plots as in (**A**) for the responses obtained in experiments with OGB-1.

We imaged GCL activity in 14 fields of view in 2 mice using the genetically encoded calcium indicator G-CaMP3 [35], and in 18 fields of view in 5 mice using OGB-1. Because GCs are closely packed, G-CaMP3 provided greater contrast than OGB-1, which may have labeled a variety of additional structures. The two types of experiment gave similar findings. We report findings obtained using both indicators and in **Figures 1** and **2** show data from G-CaMP3 experiments.

GCL cells were imaged at 28.4, 14.2 or 7.1 frames per second (fields of view 32–75 µm parasagittally by 128–150 µm mediolaterally). We selected fields of view for imaging if in resting animals, aversive stimuli evoked a visible response in putative GCs or a generalized flash in the entire field of view. In experiments with GCaMP3, putative GCs were selected based on morphology, and were defined as ring-like bright structures 4–8 µm wide with dark cores, indicative of excluded nuclear volume (**Figure 1A**; [35], [42]). In experiments with OGB-1, putative GCs were identified based on both morphology and activity, as round structures 4–8 µm wide that either were brighter than background fluorescence and/or generated at least one calcium transient during time series acquisition. For both indicators, ambiguous structures lacking these features were not analyzed.

The MF pathway to lobule IV/V is known to provide limb-associated sensory and motor information [40]. We found that during locomotion, putative GCs showed increased activity (**Figure 1A**, example traces). Locomotion onset-triggered averages (**Figure 1B**, left row) showed that the majority of GCL cells increased their activity at the onset, with increased activity persisting until the end of each period of locomotion (269 out of 285 cells, 94% for G-CaMP3; 312 out of 373 cells, 84% with OGB-1; for example see **Figure 1A**, cells #24, 33, and 60). During locomotion, signal amplitudes for all cells averaged 18±8% ΔF/F for G-CaMP3 (n = 285) and 10±4% ΔF/F (n = 373) for OGB-1, consistent with increased net excitation.

Overall, locomotion was associated with widespread activation of our selected patches in the zone A GCL, with nearly all putative GCs active within any given episode of locomotion. Locomotion-associated activity was uniformly sustained in time across GCs in G-CaMP3 measurements, but about 8% of structures labeled with OGB-1 showed transient signals, perhaps because of nonspecific uptake of OGB-1 into non-somatal processes such as mossy fibers. The remaining cells were not measurably active during locomotion (16 cells, 6% with G-CaMP3; 30 cells, 8% with OGB-1); for example see **Figure 1A**, cell 9).

To identify and count discrete GC signaling events in G-CaMP3 experiments, rising-fluorescence events were defined as transitions of >2 SD of baseline occurring from one frame to the next, and were considered to end when ΔF/F fell below +1 SD above baseline. Rising-fluorescence events occurred at a rate of 0.03±0.03 events/s during resting (285 GCs in 2 animals using G-CaMP3). The average event duration during rest was 0.66±0.32 s, a peak ΔF/F of 11±6%. Throughout most bouts of locomotion, fluorescence remained elevated, decreasing at the end, indicating that GC events were too closely spaced in time to be resolved individually. During resting periods, stimulus-triggered averages (**Figure 1B**, middle row) revealed increased activity (a change in ΔF/F of at least 2.5 times the pre-stimulus standard deviation) in most putative GCs (89%, 255 out of 285 cells for G-CaMP3; 330 of 373 cells, 91% with OGB-1). In G-CaMP3 experiments, four cells (1%) showed no detectable increase in activity either during locomotion or in response to stimuli (2%, 7 cells in case of OGB-1) and twelve cells (4%) responded to stimuli but not to locomotion (2%, 9 cells in case of OGB-1). The great majority of recorded GCL cells (245 out of 285 cells, 86%, for G-CaMP3; 313 of 373, 84% for OGB-1) responded under both locomotion and aversive stimulus (**Figure 1A and B**).

### Behavioral state-dependent GC responses to stimuli

We assessed whether responses in individual GCs to aversive stimuli were dependent on the behavioral state. In a majority of putative GCs (227 of 285 cells, 80% with G-CaMP3; 200 of 244 cells, 75% with OGB-1), stimuli evoked a response during rest but not during locomotion. Of the 227 cells, 216 cells showed increased activity during locomotion (**Figure 1B**, rows 1, 2 and 4; 183 of 200 cells with OGB-1) and 11 cells (17 cells with OGB-1) did not (**Figure 1B**, second row). A smaller number of putative GCs had detectable responses to stimuli during both rest and locomotion (28 cells, 10% with G-CaMP3; 30 cells, 12% with OGB-1), (**Figure 1B**, fourth row). A few cells responded to stimuli during locomotion but not rest (2 cells, 1% with G-CaMP3; 2 cells, 1% with OGB-1) or did not respond to stimuli under either condition (28 cells, 10% with G-CaMP3; 12 cells, 5% with OGB-1; **Figure 1B**, third row). In summary, the great majority of GCL cells responded to aversive stimuli only during rest. A summary of the distribution of response types is given in **Table 1**.

**Table 1:** Response properties of putative granule cells expressing G-CaMP3. Cells were monitored from regions within zone A that were responsive to aversive stimuli.

	Stimulus-responsive during rest only (“gated”)	Stimulus-responsive during rest and locomotion	Responsive to stimuli during locomotion, not rest	Not responsive to stimuli during rest or locomotion
**Active during locomotion**	216 (76%)	28 (10%)	1 (0%)	24 (8%)
**Not active during locomotion**	11 (4%)	0 (-)	1 (0%)	4 (1%)

To examine how the gated responses of individual GCs contributed to the local population responses in the GCL we used imaging data to calculate the mean stimulus-triggered average during resting and locomotion for all 285 GCs imaged with GCaMP3 (**Figure 2A**). Clear stimulus-induced transients could be seen during the resting state (**Figure 2A**, left), but not during locomotion (**Figure 2A**, right). The absence of a stimulus-evoked response during locomotion could have arisen from saturation of the indicator. However, calcium responses during locomotion did not reach the limits of the indicator, since locomotion-responsive GCs (n = 269, GCaMP3) showed a peak ΔF/F during locomotion (1 s moving average, excluding 0.5 s after each stimulus) of 27±15%, greater than the peak post-stimulus fluorescence occurring during locomotion (21±11%). ΔF/F reached a higher peak during non-stimulus locomotion in 224 out of 269 cells (p<10^−6^, Fisher's exact test). In many cells, the averaged stimulus-triggered fluorescence response during rest rose higher than the average fluorescence levels reached during locomotion (for example **Figure 1B**, top two rows). Thus, during locomotion the indicator was capable of generating further increases in signal. We conclude that saturation was unlikely to account for the observed suppression of responses in GCL during locomotion.

Similar results were observed for OGB-1, which lacks positive calcium-dependent response cooperativity and is therefore sensitive to an even wider range of calcium concentrations than GCaMP3 [35], [43]. To compare the overall stimulus-driven GC responses obtained using GCaMP3 and OGB-1, we calculated mean stimulus-triggered ΔF/F also for experiments performed using OGB-1 (244 putative GCs, 10 fields of view in 2 mice). As in the case of GCaMP3, a clear stimulus-triggered transient was seen during resting (**Figure 2B**, left). The responses were strongly suppressed during locomotion (**Figure 2B**, right). To address the potential effect of indicator saturation in OGB-1 imaging, we performed analysis on locomotion-reponsive cells in the same manner as for GCaMP3 experiments. The peak ΔF/F during locomotion (1 s moving average, excluding 0.5 s after each stimulus) was 11±4%, greater than the response evoked by stimuli (6±3%) during locomotion, again indicating that external stimuli did not lead to indicator saturation. Taken together, both GCaMP3 and OGB-1 results are consistent with the hypothesis that GC responses to stimuli were reduced during locomotion.

### Multiunit recording of locomotion-related responses in the granular layer

**Figure 3:**
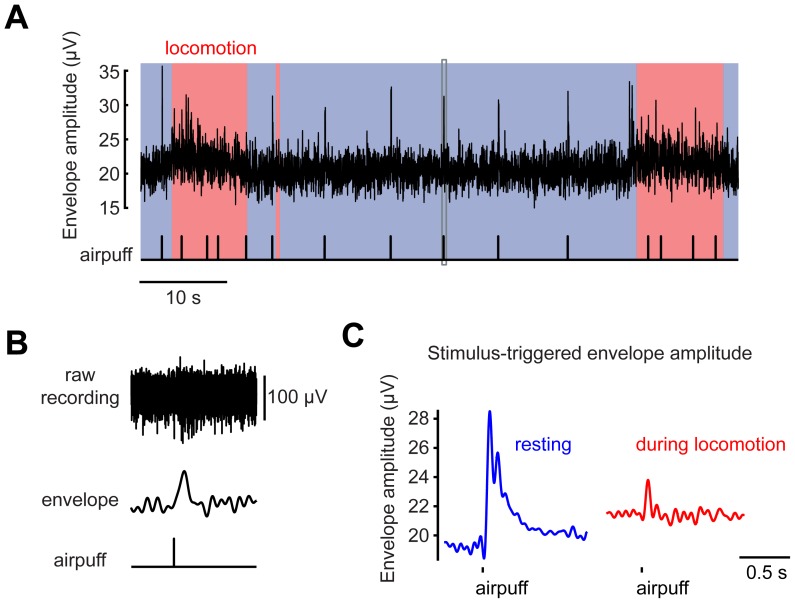
Locomotion- and stimulus-associated multiunit signals in the granule cell layer. (**A**) The signal envelope of the multiunit recordings from the granule cell layer in an awake mouse during alternating periods of rest and locomotion (pink shaded region), including airpuff stimuli (bottom tick marks). The gray rectangle indicates the region expanded in (**B**). (**B**) The raw electrophysiological recording (*top*) and the signal envelope (*middle*) associated with one airpuff. (**C**) Stimulus-triggered averages of envelope signals at rest and during locomotion.

To probe shifts in GC firing activity and stimulus responsiveness with high temporal resolution, we performed multiunit electrophysiological recording in the GCL (5 locations in 2 mice). As a measure of GCL activity we used the envelope amplitude of the 1.4–4.2 kHz response (**Figure 3A**; [9], [10]). This envelope function was bandpass-filtered at 0.01–20 Hz to represent low-frequency changes in amplitude and is an indication of total neuronal activity plus recording noise (**Figure 3B**). We selected recording locations in zone A that responded to aversive stimuli during rest.

In resting mice the envelope amplitude, reflecting recording noise and baseline neuronal activity, was 19.2±0.6 µV (35 periods of rest). Airpuff stimuli triggered a transient response lasting less than 0.5 s, reaching a peak amplitude of 28.4±4.0 µV (63 airpuffs, **Figure 3C**). All recorded regions showed an increased signal during locomotion, with an increase in envelope amplitude over rest to 21.8±0.8 µV (45 episodes of locomotion; p<0.01), indicating that local GCL activity increased with locomotion (**Figure 3A and C**). However, stimuli delivered during locomotion evoked only a small additional response (peak amplitude 23.7±3.9 µV, 61 airpuffs, p<0.01, **Figure 3C**) above the locomotion response.

To summarize the results of both calcium imaging and multiunit electrophysiology, GCL activity in zone A showed three salient behavioral state-dependent properties: transient responses to aversive stimuli during rest, increased overall activity during locomotion, and dramatically decreased responsiveness to stimuli during locomotion.

### Purkinje cell dendrite identification and recording

MF and CF projections to cerebellar cortex are thought to be co-localized based on recording [44] and tracing [45] studies and by the fact that MF and CF responses show related sensory receptive fields in PC recordings [46]. Previous studies of cerebellar c_1_, c_2_ and c_3_ zones [17], [47] and inferior olive [48] in locomoting cats showed that CF excitability varied throughout different phases of locomotion. Our recordings from GCL indicated locomotion-dependent activity and responsiveness in zone A of lobules IV/V in awake mice, suggesting that CF pathways might display the same functional features. To address this issue, we monitored the CF-driven calcium activity of local populations of PCs.

**Figure 4:**
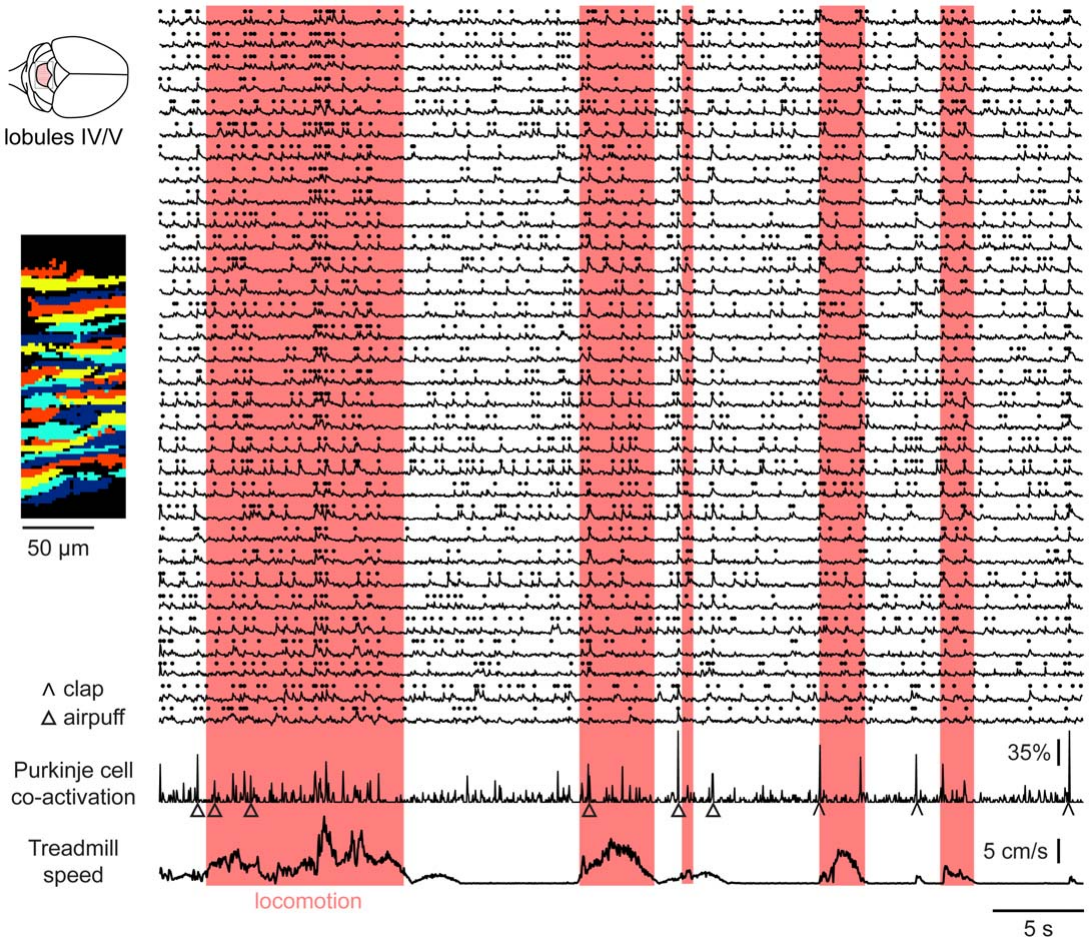
Locomotion-associated co-activation of Purkinje cell dendrites. Example data set from an awake mouse on an air-cushioned treadmill, showing signals from cerebellar Purkinje cell dendrites bulk-loaded with the calcium indicator Oregon Green BAPTA-1/AM. *Left*, field of view showing 32 dendrites identified using independent component analysis on fluorescence transients. *Center*, fluorescence traces during alternating periods of rest and locomotion. Candidate complex spike-triggered events are indicated by dots. The pink-shaded regions indicate epochs of locomotion identified from video recordings.

In two-photon movies, PC dendrites (recorded at 28.4 frames per second, fields of view ∼64 µm parasagittally by ∼256 µm mediolaterally) were apparent by their parasagittal orientation and by the occurrence of complex spike-associated calcium transients (typical peak ΔF/F_0_ = 5–20%) that spanned the entire dendrite (**Figure 4**) [14], [15]. Calcium transients were identified after high-pass filtering PC dendritic fluorescence to remove variations on time scales longer than ∼1 s and applying a filter that selected for the milliseconds-scale rise [49], [50], [51] and slower decline [15] of complex-spike-triggered calcium transients. Our procedure allowed resolution of CS-triggered events up to 5 Hz and allowed 28±6 dendrites (mean ± SD; range, 18 to 48 dendrites in 20 fields of view, 5 mice) to be monitored at once in a single field of view.

### Purkinje cell co-activation increases during locomotion

Groups of PC dendrites could encode salient information either in single-dendrite event rates [52] or in multi-dendrite synchrony [15], [19]. We therefore measured both rates and correlations during periods of rest and locomotion. In pairs of nearby PC dendrites, which predominantly receive input from different CFs [27], calcium transients often showed simultaneous onsets (**Figure 4**, top traces; see **Movie S2**), indicating the co-occurrence of complex spikes at levels far beyond those expected from chance [15].

**Figure 5:**
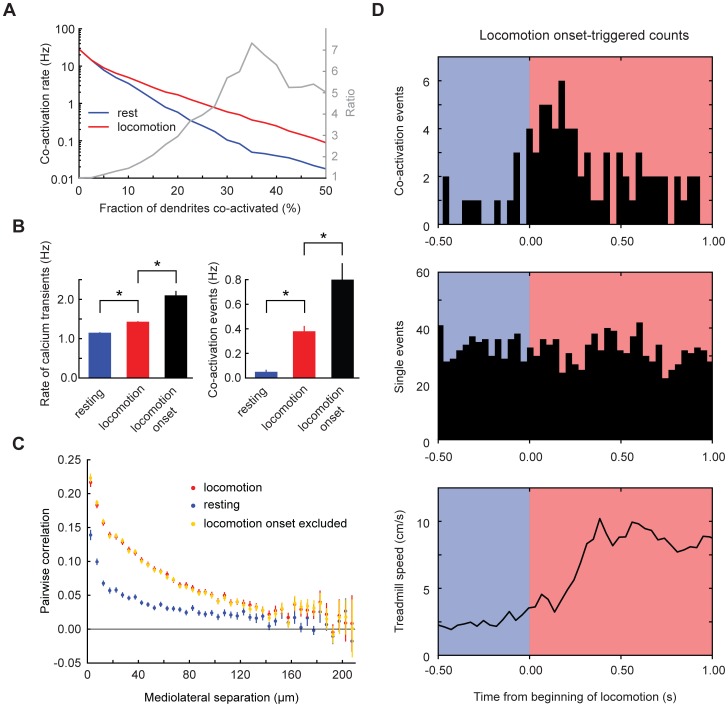
Specific increases in co-activation of nearby Purkinje cell dendrites during locomotion. (**A**) Determination of threshold for defining co-activation events. The frequency of events surpassing a given degree of co-activation was plotted for resting (blue) and locomotion (red) states. The ratio of the two quantities (black trace) shows a peak for a threshold of 35%, which therefore defines a level of maximum difference in co-activation between resting and locomotion. Co-activation events were therefore defined as events in which 35% or more dendrites were active at once. (**B**) Pooled across all experiments, average frequency of per-dendrite and multi-dendrite co-activation events as a function of behavioral state. (**C**) Pooled across all experiments, pairwise PC-PC correlation of event sequences as a function of mediolateral distance between dendrites. Error bars represent SEM. (**D**) Pooled across all experiments, locomotion-triggered histograms of co-activation events, single-dendrite-only events, and treadmill rotation speed.

Information could be encoded in the number of PC dendrites firing at once. We calculated a co-activation index, CI, defined as the fraction of PC dendrites in the field of view in which a calcium transient began during a given frame. The distribution of CI was different between periods of locomotion and rest, with the greatest difference in cumulative distribution for CI> = 0.35 (**Figure 5A**). We used this threshold to define co-activation events. All of our findings also held if we used other threshold values between 0.15 and 0.5.

In resting mice, individual PCs generated dendritic calcium transients at a rate of 1.15±0.48 Hz (mean ± SD, 937 dendrites). As the mice began to locomote, the transient rate increased modestly to 1.43±0.45 Hz (p<0.01, two-tailed paired t-test) (**Figure 5B**, left). In addition, a transient increase in rate marked the onset of locomotion (**Figure 5B**), but not the offset (data not shown). The falling time course of calcium transients was similar between rest (t_1/2_ = 0.12±0.03 s, mean ± SD) and locomotion (0.14±0.04 s; 912 dendrites), indicating that the underlying firing events were similar in both cases. Event rates were comparable to complex spike rates measured by extracellular electrophysiological recording (1.23±0.33 Hz at rest, 1.44±0.41 Hz during locomotion, 6 PCs in 3 animals) in the same mouse preparation, indicating that the imaging experiments captured most or all complex spike firing events.

More prominent than the change in the rate of calcium transients was an increase in pairwise correlation. For each pair of dendrites we calculated the event-wise correlation, a measure that is zero for independent firing sequences [15]. In both resting and locomoting animals, pairwise correlations were strongest for the shortest mediolateral separation distances (**Figure 5C**). Correlations were elevated during locomotion compared with rest (p<0.001, paired t-test) (**Figure 5C**). This elevation was significant at all mediolateral distances from 0 to 150 µm (p = 0.001 to 0.02 in individual 5-µm bins). On average, pairwise correlations declined with a mediolateral half-decay distance of 12 µm at rest and 39 µm during locomotion (**Figure 5C**). Although previous reports from anesthetized animals described discrete clusters (“microbands”) of dendrites that tended to be co-activated together over periods of ∼5 minutes [15], clustering was far less apparent during awake resting and locomotion epochs, which typically lasted for less than 10 seconds, a time scale on which clustering was made difficult by the small numbers of events, and potentially by ongoing variations in behavioral state.

Co-activation events increased over 7-fold during locomotion, from a resting rate of 0.05±0.08 events/s during rest to 0.38±0.24 events/s during locomotion (141 locomotion episodes, 16 fields of view in 5 mice) (**Figure 5B**, right). Co-activation events were particularly frequent immediately after the onset of locomotion (**Figure 5D**, top). In the first 1 s of locomotion, half of the co-activation events occurred during the first 280 ms (different from uniform distribution, two-tailed Fisher exact test, p<0.002), during which the rate was 0.8 co-activation events/s. Co-activation was also increased throughout the rest of the locomotion period (0.25±0.26 co-activation events/s, p<0.01). Throughout locomotion the rate of single-dendrite events, defined as frames in which only one dendrite initiated an event, was not elevated (**Figure 5D**, middle).

Event correlations might undergo an apparent increase without an increase in true synchrony if firing rates covary [53]. However, when we recalculated correlations during locomotion omitting the first 700 ms of each locomotion episode, the amplitude and spatial structure of correlations were unchanged (**Figure 5C**). Thus increases in co-firing events did not arise from joint variations in firing rate. Taken together, these data indicate that locomotion is associated with increases in the amount and spatial extent of complex spike synchrony in zone A, a region associated with trunk and limb representations.

**Figure 6:**
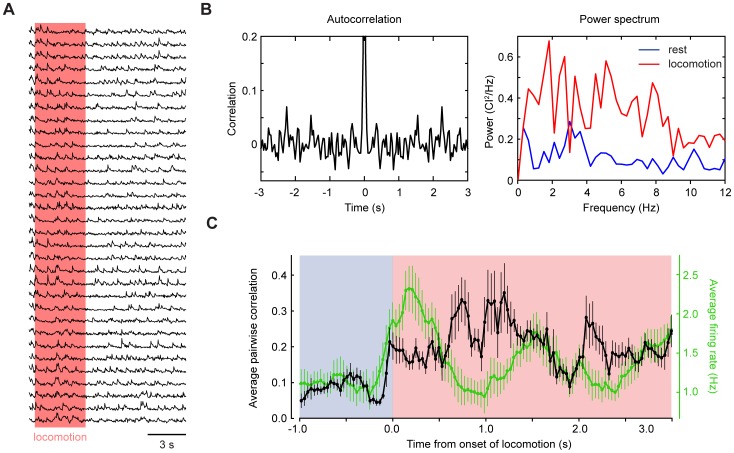
Absence of 1–10 Hz oscillation in ensembles of Purkinje cell dendrites. (**A**) Calcium signals from a typical population of imaged dendrites (**B**) Left panel, the autocorrelogram of the sum of dendritic events within the same field of view, calculated for the whole recording period. Right panel, frequency power spectra of signaling events during resting and locomotion. (**C**) Locomotion onset-triggered average firing rates and pairwise correlations show their variation around the point of behavioral change. The averages are calculated by a 387 ms sliding window. Error bars represent SEM.

Anesthetized or decerebrated animals show ensemble-level olivary activity in the form of coherent oscillations in the 3–10 Hz range [19], [54], [55]; [ though see 56]. We tested whether such oscillations occurred in our recording conditions. Dendrite-dendrite cross-correlations calculated from ensemble recordings (**Figure 6A**) showed a peak centered at zero, reflecting co-activation itself, but no additional peaks on time scales longer than 0.1 s, as would be expected for 3–10 Hz oscillation (**Figure 6B** **, left**; n = 38 recordings). The increase in synchrony during locomotion was not accompanied by narrow-band oscillation (**Figure 6B** **, right**); the average synchrony during locomotion was 0.74±0.63 CI ^2^/Hz for 1–3 Hz and 0.55±0.50 CI ^2^/Hz for 3–10 Hz (32 fields of view; not different p = 0.17, two-tailed test). To test whether increases in synchrony were coupled to CS rate, locomotion onset-triggered average firing rates and pairwise correlations were plotted (**Figure 6C**; 387 ms sliding window, 14 locomotory episodes). Pairwise correlations and firing rates increased together in the first 700 ms of locomotion, after which synchronous firing was elevated to a greater degree than firing rate. These findings suggest that under our recording conditions, olivary neurons often excite PC dendrites in unison in a rate-independent manner that is not accompanied by narrow-band oscillation.

### Aversive stimuli evoke Purkinje cell dendritic co-activation at rest, but not during locomotion

**Figure 7:**
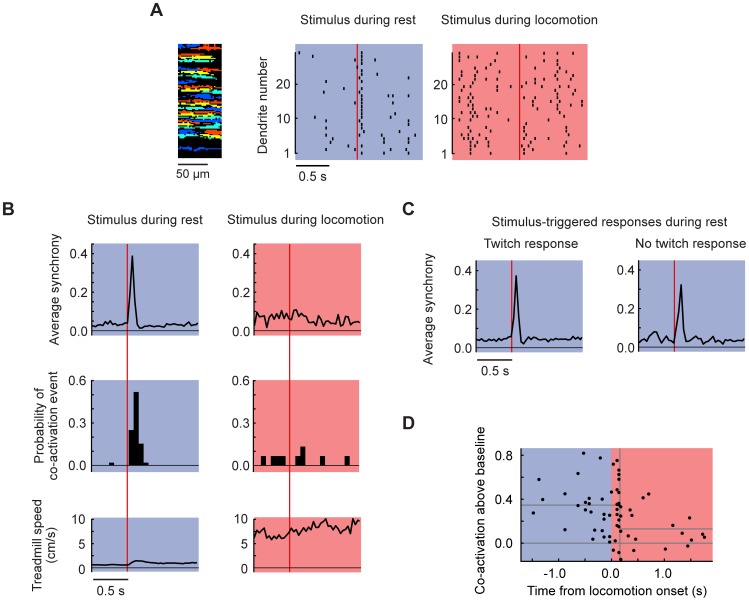
Gated co-activation of Purkinje cell dendrites by external aversive stimuli. (**A**) A raster plot from 29 Purkinje cell dendrites in response to a single clap stimulus (*vertical line*) during rest (*left*) or locomotion (*right*). (**B**) Data averaged from 5 mice from presentations of multiple stimuli in which the animal did not begin to move (*left*; 52 stimuli in 16 fields of view) or was already locomoting (*right*; 15 stimuli, 9 of the same fields of view). *Top*, average co-activation plotted frame by frame. *Center*, the probability of a co-activation event. *Bottom*, treadmill rotation speed. (**C**) Average synchrony response in resting animals divided according to whether the stimulus triggered a visible twitch. (**D**) Fraction of co-activated dendrites in the first 3 frames after a stimulus, plotted relative to the time of the nearest locomotion onset for all fields of view. Stimuli were given at various times distributed along the horizontal axis. Plotted values were corrected by subtracting the co-activation fraction (which was higher during locomotion) occurring at each time point when no stimulus was given. A break point (*vertical line*) 0.14 s after locomotion onset was identified by deviance minimization.

Past research in anesthetized rats [57] and cats [40] reported complex spike responses in zone A to limb and trunk stimuli. We therefore measured PC dendritic responses to aversive stimuli (**Figure 7A**). In resting animals, we used electrophysiological measurements to monitor the occurrence and precise timing of complex spike responses. The latency to response from the onset of the stimulus was 53±20 ms for airpuffs and 49±13 ms for claps. In recordings from stimulus-responsive PCs, the complex spike probability within 150 ms was 0.42±0.33 for airpuffs and 0.33±0.32 for claps, comparable to previously reported event-associated complex spike probabilities [23], [44], [58]. Probabilities were correlated (airpuff vs. clap r = +0.6) and both types of stimulus could provoke locomotion, suggesting that responses were not specific to a particular sensory modality. For further analysis of locomotion-dependence, results from the two types of aversive stimulus were pooled.

Since co-activation was high during movement, it was possible that responses to stimuli were purely a reflection of locomotion-related activity. To test this, we examined responses in cases where stimuli were not followed by locomotion. Under this condition, over 35% of PC dendrites within all fields of view were co-activated within 3 frames (105 ms) after the stimulus (**Figure 7B**, left panel) with an average CI of 39±30% (mean ± SD, **Figure 7B**, left panel, 52 stimuli). We further subdivided no-locomotion trials into cases in which the mouse did or did not give a startle response. In this classification, non-startle trials were those in which muscle activity was below the level of visible detection. The stimulus-triggered peak co-activation values (**Figure 7C**) were 42±33% (32 stimuli) for startle responses and 34±31% (17 stimuli) for non-startle responses (not significantly different, p = 0.4, two-tailed t-test). Thus aversive stimuli triggered complex spike co-activation responses, with or without the occurrence of detectable movement.

The independence of co-activation responses from subsequent movement suggested that responses reflected a signal that was in nature preparatory for action. In an alternate possibility, the responses could be purely sensory [57], in which case they would occur whether or not the animal was locomoting. However, co-activation was high in cases where stimuli were followed by locomotion (CI = 28±25%, 27 stimuli, p = 0.1, not shown) and nearly absent when stimuli were applied during locomotion (CI = 11±13%, 15 stimuli for the same fields of view, p<0.01, **Figure 7B** right panels and **Figure 7D**). The conditional nature of the response is consistent with previous research on gating of responses, in which CF responses to touch are greater during rest and for unexpected stimuli than during action [59], [60]. To summarize, strong PC co-activation was evoked by several kinds of action-triggering stimuli, but only if locomotion had not already begun.

Reduced PC dendritic responsiveness during locomotion could potentially arise from recent firing history. In this scenario, responses in a specific group of neurons would be less responsive after firing recently, a form of relative refractory period. To test the contribution of this possibility to the observed responses we examined responses to aversive stimuli during all non-locomotory periods. We classified each dendrite on a trial-by-trial basis into cases where it fired or not in the 500 ms before the stimulus. We then calculated co-activation statistics for recently-firing dendrites and recently-silent dendrites. In dendrites that fired recently, the mean CI was 35±33%, compared with 30±30% (52 stimuli) for recently-silent dendrites (p = 0.7, 52 stimuli). Therefore a dendrite-specific decrease in responsiveness cannot account for the suppression of PC co-activation during locomotion.

Past work on gating suggested that the suppression of PC responses to stimuli might occur sharply in time. To identify the timing of such a gating signal, the maximal co-activation in the 3 frames following a stimulus was plotted relative to the time of the nearest start of locomotion (**Figure 7D**). To correct for elevated CI during locomotion, the average CI was subtracted for the corresponding time from locomotion episodes where no stimulus was given. The CI decreased soon after the start of locomotion. To define the transition time, a break point was found that minimized the variation in CI on either side of the break (deviance minimization; see **Materials and Methods**). This break occurred at t = +140 ms (half-maximal range +110 to +380 ms) after the start of locomotion, with a CI of 35±23% before (n = 43) and 13±17% (n = 19) after the break. Thus, in the CF pathway, “gating-off” (i.e. suppression) of co-activation responses to stimuli occurs within a fraction of a second at the moment of locomotion's onset. In summary, our PC recordings demonstrate two types of locomotion-related complex spike co-activation signals in zone A: triggers of locomotion and ongoing signals throughout locomotion. Furthermore, aversive stimuli co-activate many PC dendrites at once, but only during rest.

### Increased activity in molecular layer interneurons with locomotion and aversive stimuli

Stellate and basket interneurons are driven by GCs, suggesting that their activity would reflect the overall dynamics of the GC population. Interneurons also receive excitation from CFs when they are multiply activated at once via glutamate spillover [61]. We therefore expected interneurons to show signals that reflected a combination of GC activity and PC dendritic co-activation. As a test, in the molecular layer we imaged (14.2 frames per second, fields of view ∼128 µm parasagittally by ∼256 µm mediolaterally) the somata of molecular layer interneurons, identified by their size and location above the PC layer [13]. Regions of interest were manually drawn around interneurons, which were bright and typically spaced tens of microns from one another (**Materials and Methods**). Imaging was also used to place extracellular recording electrodes for single-unit recording.

**Figure 8:**
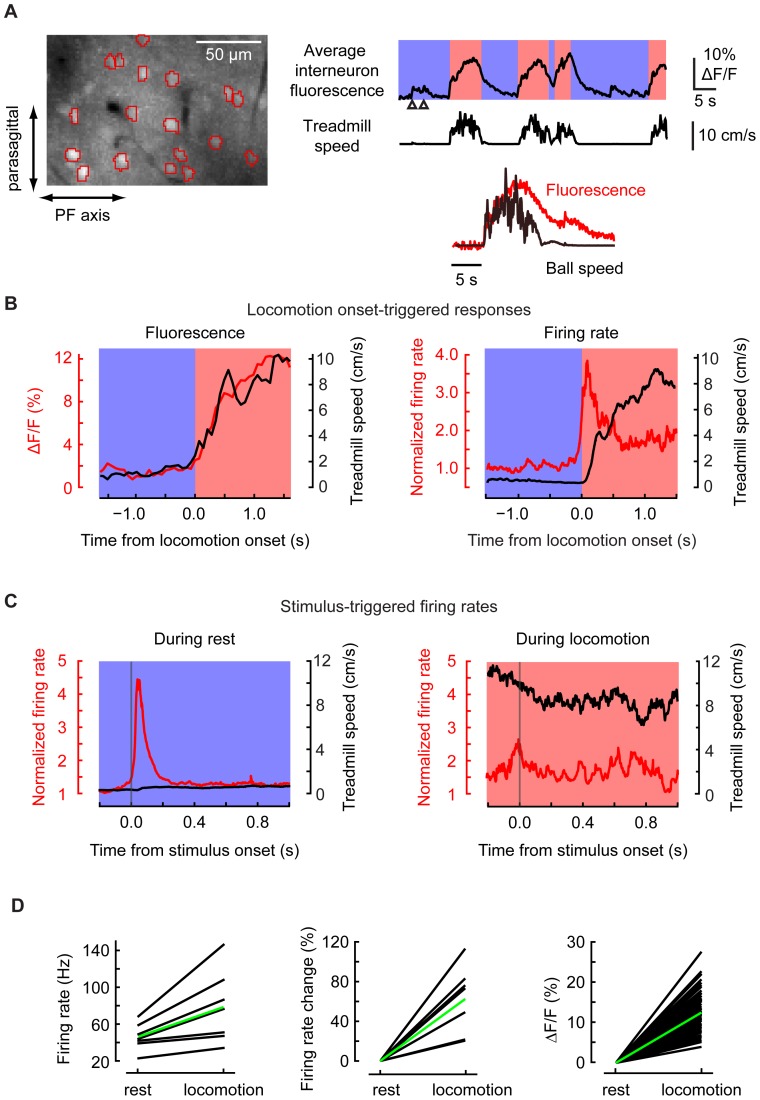
Locomotion and stimulus-dependent activity in molecular layer interneurons. (**A**) *Left*, an example field of view showing 18 molecular layer interneurons. *Right*, averaged fluorescence from all interneurons during alternating periods of resting (blue shading) and locomotion (pink), including two airpuff stimuli (arrowheads). Inset, movement onset-triggered averages of treadmill rotation and fluorescence, taken from 7 episodes of movement. (**B**) Locomotion onset-triggered averages as measured by (left) fluorescence and (right) single-unit extracellular electrophysiological recording in the molecular layer. For both cases, the average treadmill speeds are shown in black. (**C**) Stimulus-triggered average responses to claps in mice at rest (52 claps, 7 cells) and during locomotion (7 claps, 4 cells) taken from extracellular recording. Vertical lines indicate the time of the clap. (**D**) Relative increases in interneuron activity during locomotion as depicted by electrophysiology (7 interneurons, left and middle panels) and imaging (75 interneurons, right panel). In each panel, dark lines indicate the activity of individual interneurons and green line the averaged activity over all interneurons.

To measure locomotion- and stimulus-associated changes in interneuron activity, 75 molecular layer interneurons were imaged in 5 fields of view in 3 mice (13 to 18 interneurons per field of view). All interneurons showed elevations in intracellular calcium concentration during locomotion (ΔF/F = 13±5% compared with rest, averaged over all the locomotion episodes, mean ± SD, n = 75; **Figure 8A**). Fluorescence began to rise shortly after the onset of locomotion (2 SD above baseline starting +103±168 ms after locomotion onset; mean ± SD, n = 53, **Figure 8B** left) and was elevated for the duration of locomotion. Individual interneuron traces were similar to the average, and were very similar to one another during epochs of locomotion. Excluding the first and last 1.5 s of the locomotion epoch, correlation coefficients were +0.27±0.36 (mean ± SD, 777 pairs), significantly higher than pairs of cells recorded during different epochs (+0.01±0.21; one-tailed t-test, P<0.001). Therefore locomotion-related signals in interneurons are consistent with the combined action of MF activity and CF synchrony.

We used single-unit recording to test whether interneuron responses to aversive stimuli were also dependent on behavioral state (**Figure 8C**). In resting animals, claps triggered transient firing starting at +21±22 ms (mean ± SD) after the stimulus (n = 52) and lasting up to 88 ms after the stimulus, reaching an average firing rate of 129±67 Hz and a peak (2 consecutive ISIs) of 261±136 Hz. Firing rates increased from 46±15 Hz during rest to 78±39 Hz during locomotion, consistent with the fluorescence measurements (**Figure 8D**). During locomotion, claps did not evoke a response (average rate 82±25 Hz, 7 claps; not different from rates during locomotion without an aversive stimulus, p = 0.6).

Because both GC activity and interneuron activity both increased, the net effect of MF pathway activation on simple spike (SS) firing might be excitatory or inhibitory. To measure the effect directly, we made single-unit extracellular recordings of SS activity from five PCs. Resting firing rates ranged widely, from 25 Hz to 143 Hz, and the average firing rate was nearly unchanged between rest (81±23 Hz, mean ± SEM) and running (95±16 Hz). At the level of individual PCs, changes in firing rate varied inversely with resting firing rates (rank correlation r = −0.8): PCs with low resting rates (25 Hz and 31 Hz) accelerated during locomotion (to 52 Hz and 65 Hz), while the faster-firing PCs did not change (143 Hz to 144 Hz). The two slowest-firing PCs also increased firing rate in response to stimuli, whereas high-firing cells showed no change or a small decrease. These data suggest that during running activity and external input, SS activity is redistributed across the PC population.

## Discussion

In a region of cerebellar cortex that represents trunk and limb sensory/motor information, we have described concerted behavior-dependent changes in the activity of the mossy fiber (MFs) and climbing fiber (CF) pathways. We found that locomotion was associated with large-scale shifts between two major regimes of activity for both pathways: (1) As locomotion began, GC activity and complex spike co-activation rose transiently. These rises were sharp at the onset of locomotion and were followed by a sustained plateau. Shortly after locomotion onset, aversive stimuli became ineffective at triggering additional increases in GC or complex spike activity. (2) In resting mice, CF co-activation was low and GCs were less active than during periods of locomotion. Aversive stimuli that could trigger locomotion activated widespread transient increases in GC activity and increases in complex spike co-activation across groups of PC dendrites. Taken together, our results demonstrate rapid shifts in activity patterns and responsiveness of the MFs and CF pathways, modulated by the locomotory state of the mouse.

GCs are not fixed in their representation of events. Our studies are the first to report activity from local populations of putative individual GCs in awake, behaving mammals. Multiunit GC activity has been monitored previously during motor behavior [9], [10]. In grooming, eating, and exploring rats, activity is elevated throughout behavior [9]. In primates performing expectation-based lever pressing tasks, local field potential oscillations in different locations in the granular layer become synchronized during task performance [10]. Based on the prior studies, GC layer activity is elevated and synchronized during motor behavior. Our research probes the extent of these changes in GC populations from behaving animals.

Theoretical analysis of anatomy has predicted a sparse code in which a small fraction of cells are active at any one time [62], [63], consistent with later intracellular recording studies in anesthetized rats [5] and awake fish [64] (though not decerebrated cats [65]). Our findings show that over periods of locomotion lasting a few seconds, elevations in GCL activity in awake animals can recruit a large fraction of cells at some point during that interval. Widespread activation has been observed in zebrafish GCs during optokinetic reflex behavior (S. Sylvester, K. Daie, M. Lee, and E. Aksay, 2011, *Soc. Neurosci. Abstr.* 922.12). Widespread activation is also reflected in our interneuron recordings, whose time courses are uniformly similar within a field of view. Interneurons might play a broad integrative role in which they inhibit PCs by an amount that scales with the summed afferent activity over many MFs and CFs.

Calcium imaging methods are limited in their ability to determine the extent to which GCs are co-activated during behavior. In both GCs and interneurons, somatic calcium clearance of ∼1 s limits temporal resolution [13], [66]. In the future, cell type-specific expression of genetically encodable calcium indicators can potentially facilitate imaging in processes, where clearance times are faster. In this case, a limiting factor is the off-response of probes: at mammalian temperatures, GCaMP3's off-response to calcium has a time constant of 0.25 s, a parameter that is targeted for future improvement (X. R. Sun, Y. Lampi, T. Friling, B. Kuhn, E. Schneider, S. S. Lin, S. S.-H. Wang, 2011, *Soc. Neurosci. Abstr.* 840.03). For the same reasons, the GC response categories we report represent a necessary simplification compared with the temporal detail observable in electrical recordings from MFs ([6]; see Figs. 7 and 8; see also [67]). Improved temporal resolution will help distinguish the contributions of MF activation and CF synchrony, as well as clarify issues relating to GC population coding.

Our inferences of individual GC and interneuron activity are also limited by the mechanisms by which calcium signals arise and are detected. GC calcium signals arise from both action potentials and from dendritic calcium entry driven by NMDA receptor activation [68], [69], raising the possibility that some fraction of the observed optical signal arises from synaptically-mediated calcium entry. Recent recordings from head-fixed locomoting mice show gating of stimulus-evoked GC spiking responses as measured by whole-cell patch clamp recording (K. Powell, T. Branco, I. Duguid, and M. Häusser, 2011, *Soc. Neurosci. Abstr.* 922.02). The interneuron activity we observe reflects shifts in GC firing (and also CF activation; see Szapiro and Barbour (2007)).

Our results broaden the principle of functional convergence of MF and CF pathways. PC simple spikes (reflecting net excitation via MF activation of GCs and interneurons) and complex spikes (reflecting CF activation) have previously been observed to respond to similar or related touch fields [46], consistent with anatomical co-mapping of MFs and CFs in cerebellar cortex [45]. In our results, the same cerebellar circuitry can modulate action (as in activity generated during locomotion) or respond to stimuli that trigger action (as in aversive stimuli). MF and CF collaterals and PC axons furthermore converge on common deep nuclear targets for downstream readout [28], [70]. We suggest that under a range of behavioral conditions CF synchrony may provide ongoing corrective drive to guide action appropriately, depending on whether an action is being initiated or adjusted.

Cerebellar processing eventually converges on the deep nuclei, which stand at the terminal apex of a highly organized three-part olivocerebellar circuit. A second part is the PCs with their specific simple and complex spike activity. We monitored complex spike-evoked calcium signals in PC dendrites, which reflect activity in the third part of the circuit, the inferior olive. The inferior olive, the PCs, and the deep nuclei observe a precise anatomical linkage in which each olivary neuron innervates an average of 5–6 parasagittally-aligned PCs via CFs [27], [71], [72], while also sending an axon collateral to innervate a deep nuclear target. CF, olivonuclear, and corticonuclear axons project in a maplike fashion: neighboring neurons in one region (olive, cortex, or deep nucleus) innervate neighbors in the other two regions. In this “microzone” arrangement, synchronized olivonuclear excitation followed by PC inhibition (and a pause) would have a focused effect on a specific part of the deep nuclei.

Olivary synchrony has been suggested to be important in driving a 1–10 Hz oscillation that serves as a form of clock [55]. Under our experimental conditions, climbing fibers do not show evidence of this oscillation, raising the question of what function is served by non-oscillatory olivary synchrony. Isolated synchronous events are potentially detected by multiple mechanisms. First, olivary synchrony may distinguish event-specific firing from ongoing background activity [15], [25]. In analogy to the CF role in instructing plasticity at PC synapses, CF co-activation could provide an instructive signal via olivonuclear collaterals to drive long-term plasticity at MF-nuclear or olivonuclear synapses [29]. Second, co-activation of CFs triggers a post-complex-spike pause in many PCs at once, which could facilitate deep nuclear firing by suddenly removing inhibition [73]. Finally, molecular layer interneurons can be excited by multiple CFs [61]. In these three ways the inferior olive might provide co-activation-based instructive and/or modulatory signals distinct from those received by individual PCs.

Encoding of touch to a limb by CFs has previously been reported to depend in a detailed manner on the animal's behavioral state [74]. Sensitivity can change in less than a second, varying even as a function of time during the walking cycle, representing a shift in influence between ascending (spinal cord) and descending (forebrain) pathways [30]. We found reduced responsiveness to clap sounds as well as to touch delivered via airpuffs, indicating that the reduction in sensitivity was not restricted to a single sensory modality as previously suggested [75]. Our use of auditory stimuli also argues against peripheral mechanisms such as reduced sensory stimulus during movement. Our findings reveal state-dependent representations that are not specific to touch but instead are likely specific to an action, in this case locomotion.

In addition, our findings report the strongest modulation yet demonstrated of CF synchrony by behavioral state. We observed state-specific co-activation of a large percentage of the PCs (>35%), especially at the onset of locomotion and in response to stimuli during rest. We previously demonstrated high levels of co-activation in imaging and in line scans (2 ms) [15]. Electrophysiologically observed complex spike synchrony occurs on the time scale of 1 to 10 milliseconds [18], [19], [20], [76], [77]. In PCs separated by 0.2–2 mm, weak complex spike synchrony has been observed associated with licking [19] and locomotion [76]. In our experiments, which surveyed nearly every PC dendrite within a field of view, increases in co-activation were an order of magnitude higher.

Our results from PCs contrast with some previous imaging studies in awake mice. One study [52] reported a 3-fold increase in complex spike firing rates but only a 20% increase in pairwise correlation during locomotion, whereas we found modest changes in rate and a larger increase in correlation. The discrepancy could arise from incomplete event detection by Flusberg et al. since their baseline calcium transient rates were ∼0.2 Hz, lower than known CF firing rates. Later imaging studies from the same group, done in head-fixed mice on an exercise ball [26] and in freely-moving mice [78], gave results more consonant with ours, with moderate increases in single-cell transient rates and large increases in pairwise correlation during locomotion. It should be noted that our apparatus can be used in conjunction with a virtual-reality system to investigate spatial navigation [79] and to achieve reliable classical eyeblink conditioning (A. Giovannucci, F. Najafi, A. D. Kloth, J. F. Medina, S. S.-H. Wang, 2011, *Soc. Neurosci. Abstr.* 183.19), indicating that mice do not experience undue stress while on the treadmill.

The simultaneous behavioral gating of stimulus-related GC activity and Purkinje cell co-activation suggests that upstream inputs controlling CF and MF activity are redirected together to influence action in a coordinated manner. One candidate for a common upstream trigger is serotonergic neurons of the dorsal raphé. These neurons project to the inferior olive, to the granular layer [80], and to nuclei that give rise to major MF projections. Dorsal raphé neurons fire during locomotion [81] and show rapid behavior-specific modulations in firing rate on the scale of tens of milliseconds [82]. Serotonergic neurons project to the inferior olive and to nuclei that give rise to major MF projections. Activation of this trigger could rapidly redirect alternate streams of input to the CF and MF systems to influence cerebellar output and learning. Such a mechanism would be an example of coordinated regulation of multiple brain pathways by a core neuromodulatory system. Other possible controllers of gating include pathways of spinal and neocortical origin. Whatever the source of gating control, our results show that CF synchrony and GC activity can act in a state-specific manner so that at different times, the same circuitry can rapidly shift modes to focus on either external or internal signals.

## Supporting Information

Movie S1 **Performance of motion correction algorithm.** A fluorescence movie is shown before (left side) and after (right side) correction for brain motion. The field of view is 64 µm (parasagittal) by 256 µm (mediolateral) for the movie on the left, and 60 µm by 248 µm for the movie on the right.
(MOV)

Movie S2 **Purkinje cell dendritic activity during behavior.** ΔF/F activity of 32 Purkinje cell dendrites (left side) was monitored during the animal's spontaneous behavior (right side). ΔF/F activity was coded in the shades of red and behavior was captured by an IR camcorder. The field of view is 64 µm (parasagittal) by 256 µm (mediolateral). Note that the field of view is elongated in the parasagittal direction for display purposes.
(AVI)

Movie S3 **Interneuron activity during behavior.** ΔF/F activity of 8 interneurons (left side) is monitored during mouse's spontaneous behavior (right side). ΔF/F activity was coded in the shades of red and behavior was captured by an IR camcorder. The field of view is 56 µm (parasagittal) by 256 µm (mediolateral). Note that the field of view is elongated along the parasagittal direction for display purposes.
(AVI)
